# Transgenerational inheritance of impaired larval T cell development in zebrafish

**DOI:** 10.1038/s41467-020-18289-9

**Published:** 2020-09-09

**Authors:** Norimasa Iwanami, Divine-Fondzenyuy Lawir, Katarzyna Sikora, Connor O´Meara, Kohei Takeshita, Michael Schorpp, Thomas Boehm

**Affiliations:** 1 Department of Developmental Immunology, Max Planck Institute of Immunobiology and Epigenetics, Stuebeweg 51, 79108 Freiburg, Germany; 2 RIKEN SPring-8 Center, Sayo, Hyogo, 679-5148 Japan; 3 Present Address: Center for Bioscience Research and Education, Utsunomiya University, 350 Mine-machi, Utsunomiya, Tochigi, 321-8505 Japan; 4 Present Address: Institute of Zoology, University of Cologne, Zülpicher Str. 47b, 50674 Köln, Germany

**Keywords:** Embryology, Epigenetic memory, DNA methylation, Immunology, Epigenetics

## Abstract

Evidence for transgenerational inheritance of epigenetic information in vertebrates is scarce. Aberrant patterns of DNA methylation in gametes may set the stage for transmission into future generations. Here, we describe a viable hypomorphic allele of *dnmt1* in zebrafish that causes widespread demethylation of CpG dinucleotides in sperm and somatic tissues. We find that homozygous mutants are essentially normal, with the exception of drastically impaired lymphopoiesis, affecting both larval and adult phases of T cell development. The phenotype of impaired larval (but not adult) T cell development is transmitted to subsequent generations by genotypically wildtype fish. We further find that about 200 differentially methylated regions in sperm DNA of transmitting and non-transmitting males, including hypermethylated sites associated with *runx3* and *rptor* genes, whose reduced activities are associated with impaired larval T cell development. Our results indicate a particular sensitivity of larval T cell development to transgenerationally inherited epimutations.

## Abstract (web summary)

Evidence for transgenerational inheritance of epigenetic information in vertebrates is scarce. Here the authors report that homozygous dnmt1 mutant zebrafish are essentially normal, with the exception of impaired lymphopoiesis, with impaired larval (but not adult) T cell development being transmitted to subsequent generations by genotypically wildtype fish.

## Introduction

The transgenerational inheritance of epigenetic information is an attractive mechanism by which the phenotypic consequences of exposure of parents to certain environmental conditions could be transmitted to the next generation^1,2^. Although this phenomenon is well described in yeast and plants, and in some invertebrates, such as *D. melanogaster* and *C. elegans*, the presence of transgenerational inheritance in vertebrates is unclear^3^. Among the mechanisms that are known or suspected to be involved in mediating the propagation of epigenetic information to subsequent generations, the process of enzymatic DNA methylation is of particular interest^4–6^. In the context of epigenetic inheritance, the induction of aberrant methylation patterns in gametes may set the stage for transmission into future generations^1,2,7^. With respect to the fate of germ cell DNA methylation patterns during early embryogenesis, important differences exist among vertebrate species. During the expansion of mouse primordial germ cells of mammals, global loss DNA methylation occurs^8,9^; a similar reversion to the native ground state is observed during early stages of human development^10,11^. Thus, the global erasure of methylation patterns in mammals constitutes a strong barrier to epigenetic memory mediated by altered patterns of DNA methylation^12^. By contrast, the dynamics of DNA methylation during early stages of development in zebrafish are very different and may be conducive to transgenerational inheritance. The methylome of zebrafish sperm is significantly hypermethylated when compared to that of oocytes; during the course of development, the methylome of the oocyte is gradually reprogrammed to a pattern that is similar to that of the sperm, such that by the midblastula stage, the embryo´s methylation pattern is virtually identical to the sperm methylome^13,14^. Of note, the reprogramming process does not encompass the entire genome, but depends, at least in part, on the activity of so-called placeholder nucleosomes^15^. The stability of the paternal methylome throughout development^16,17^ raises the possibility that aberrations in the DNA methylation pattern of sperm DNA might be transmitted to the next generation.

Methylation of cytosines in DNA is established by de novo methylases Dnmt3a and Dnmt3b, whereas its propagation after DNA replication and repair depends on the maintenance methylase Dnmt1, which recognizes the hemi-methylated DNA duplex and copies the methylation pattern of the parental strand to the newly synthesized DNA strand^18^. Mice lacking *Dnmt1* die at around day 9.5 of embryonic development^19^; likewise, zebrafish homozygous for a mutant *dnmt1* allele predicted to encode an enzyme with impaired function of the catalytic domain die at 8 days post fertilization (dpf)^20^. These findings attest to central cellular function of Dnmt1^18^, but conceal a possible tissue-specific function of this protein in the adult organism. However, evidence for tissue-specific requirement of *Dnmt1* has come from conditional mutants in mice (for instance, see ref. ^21^), and the phenotype of zebrafish *dnmt1* morphants^22^. However, these models fall short of providing a pan-organismic view of DNMT1 function in vertebrates.

Here, using a forward genetics approach^23^, we describe the identification and characterization of viable recessive allele of zebrafish *dnmt1*. Our results provide evidence for the transgenerational inheritance of aberrant DNA methylation patterns associated with impaired larval T cell development.

## Results

### Identification of a recessive viable allele of *dnmt1*

**Fig. 1:**
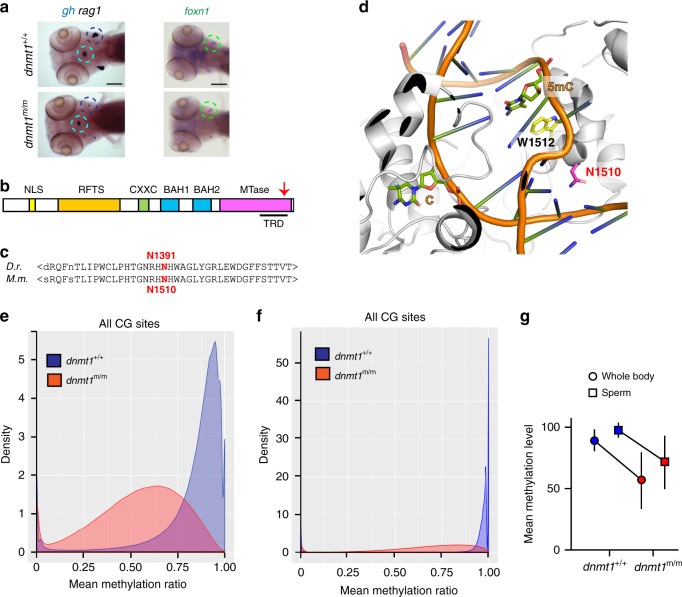
Characterization of a hypomorphic allele of *dnmt1*. **a** Impaired larval T cell development in mutant fish. Diagnostic whole-mount RNA in situ hybridization pattern ^23^in wild-type and *dnmt1* mutant fish at 5 dpf using *rag1* (thymus encircled in black), and *gh* (hypophysis encircled in blue) specific probes (left panel); hybridization pattern for *foxn1*, a marker of thymic epithelium^59,60^ (right panel). Scale bar, 100 μm. Panels are representative of 25 animals each. **b** Schematic of functional domains in the dnmt1 protein (not to scale); NLS, nuclear localization signal; RFTS, replication foci targeting site; CXXC, cysteine-rich domain; BAH, bromo-adjacent homology domains 1 and 2; MTase, catalytic domain. Arrow, approximate position of the amino acid replacement in the target recognition domain, TRD. **c** The asparagine residue mutated in the *dnmt1*^t25501^ allele occurs in an evolutionarily conserved region of the enzyme. **d** In the structure of the mouse Dnmt1 protein in complex with a hemi-methylated substrate, close apposition of the mutated asparagine residue to the substrate DNA in the catalytic site is observed^26^ (PDB ID: 4DA4). **e**, **f** Hypomethylation of cytosine residues in CpG dinucleotides of DNA extracted from whole body of 18 dpf larvae (**e**) (*n* = 3) and sperm of adult homozygous mutants (**f**) (*n* = 3). In (**e**) and (**f**), density refers to the fraction of CG sites with a particular methylation ratio. **g** Mean methylation levels of CG dinucleotides for DNAs shown in (**e**) and (**f**). Values shown represent mean ± mad; *n* = 3. Source data are provided as Source Data file.

In a forward genetic screen for aberrant T cell development, we identified a large number of recessive mutations, all characterized by reduced numbers of *rag1*-expressing immature thymocytes in larvae 5 days after fertilization (dpf)^23,24^. The mutation in the IY071 line^23^ (Fig. 1a) affected the gene encoding the maintenance DNA methyltransferase DNMT1. The recessive viable *dnmt1* allele (*dnmt1*^t25501^) exhibits a missense mutation (N1391K) in the target recognition domain (TRD) of the catalytic domain of the enzyme (Fig. 1b)^23^; since a previously identified *dnmt1* mutant allele is embryonic lethal^20^, we consider the *dnmt1*^t25501^ allele to be a hypomorph. The mutation in *dnmt1*^t25501^ occurs in an evolutionarily conserved region of the protein (equivalent to N1510K in the mouse protein) (Fig. 1c), and affects an amino acid residue whose side chain in the mouse protein projects towards the major groove of the substrate DNA^25,26^ (Fig. 1d).

### Hypomethylation of DNA in *dnmt1* mutants

Fish homozygous for the N1391K mutation exhibit drastically reduced levels of cytosine methylation in 5´-CpG dinucleotides of genomic DNA. In DNA extracted from wild-type whole fish at 18 days after fertilization (dpf), 89.08% (median; median absolute deviation [mad] 8.79%) of CpG dinucleotides are methylated whereas the methylation levels in mutants are much lower reaching a mere 56.72% (median; mad 23.04%) (Fig. 1e). The methylation levels in sperm DNAs are generally higher than in somatic tissues, reaching 97.62% (median; mad 3.09%) in the wild-type situation; in *dnmt1*^m/m^ males, in which germ cells develop in the absence of zygotically provided dnmt1 protein, methylation levels fall to 71.81% (median; mad 21.52%) (Fig. 1f). The extent of hypomethylation is more pronounced in DNA extracted from whole body as compared to mutant sperm, falling to 63.6% and 73.6% of wild-type values, respectively (Fig. 1g). Remarkably, despite the dramatic hypomethylation of somatic DNA, *dnmt1* hypomorphs are viable, making this zebrafish strain an unprecedented model with which to explore the physiological consequences of low DNA methylation levels in an organismic context.

### Hematopoietic abnormalities in *dnmt1* mutants

**Fig. 2:**
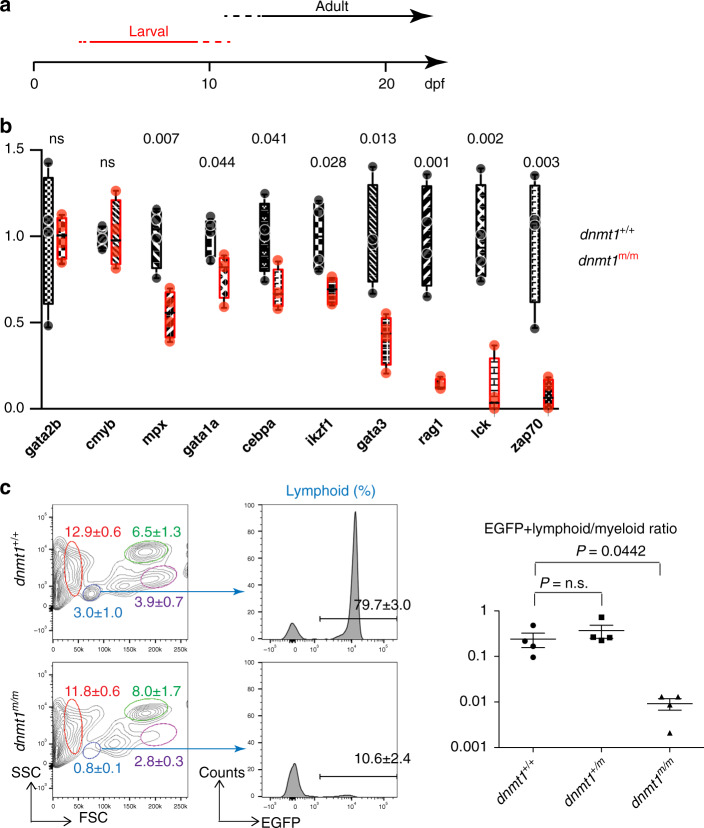
Hematopoietic abnormalities in *dnmt1* mutant fish. **a** Schematic indicating the two waves of T cell development in zebrafish, operationally referred to as larval and adult phases; the larval phase depends on the activity of the ikzf1 transcripton factor^28,46^. In the forward genetic screen, fish were analyzed at 5 dpf. **b** Expression patterns of selected genes associated with hematopoietic development in 5 dpf G2 embryos. Levels were determined by qPCR and normalized to the levels of *actb1* (*n* = 4; mean; whiskers represent maximum and minimum values; individual data points are indicated). **c** Adult lymphopoiesis fails in mutant fish. Flow cytometric analyses of whole kidney marrow (WKM) cells^47^ of wild-type and *dnmt1* mutants, both transgenic for an *ikzf1-EGFP* reporter^27,28^ (left panels). Circles denote different blood cell populations in adult wild-type fish: red, erythrocytes; blue, lymphocytes; magenta, precursors; green, myelomonocytes. Percentages (mean ± SD) of each population in WKM preparations are indicated (*n* = 4, for both genotypes; left panels). Analysis of EGFP-positive cells in the lymphocyte gates; percentages of positive cells are indicated in the histograms (*n* = 4, for both genotypes; middle panel), and enumerated (right panels). FSC, forward light scatter; SSC, side light scatter. Source data are provided as Source Data file. For b, c, unpaired two-tailed *t* test with Welch´s for unequal variance and Bonferroni´s correction for multiple tests.

Next, we examined the hematopoietic system in *dnmt1*^m/m^ animals during larval and adult stages (Fig. 2a). At 5 dpf, no changes were recorded for the expression levels of markers for hemogenic endothelium (*gata2b*) and haematopoietic stem cells (*c-myb*). Slightly reduced levels were found for genes indicative of erythroid (*gata1a*) and myeloid (*cebpa*) lineages, although expression of *mpx* as a marker of mature myeloid cells was more substantially reduced (Fig. 2b). At this stage of development, the most drastic changes in expression were seen for genes associated with T cell development (*lck*; *zap70*) and for genes whose expression is associated with, but not restricted to, developing T cells (*rag1; ikzf1*; *gata3*) (Fig. 1a; Fig. 2b). Next we examined the status of adult hematopoiesis in whole kidney marrow. Among the cell populations discernable by their light-scatter characteristics, the most consistent reduction was seen for lymphocytes, whereas erythroid and myeloid cell populations were not affected (Fig. 2c). In order to substantiate this conclusion, we introduced an *ikzf1:egfp* transgene^27^ into the IY071 line; in adult fish, the reporter marks T and B cells. As suggested by the aberrant flow cytometric profile, the lymphocyte population is greatly diminished in *dnmt1*^m/m^ fish (Fig. 2c). Collectively, our results suggest the lymphopoietic capacity of adult *dnmt1*^m/m^ animals is drastically impaired, indicating that the genetically separable waves of larval and adult T cell development in zebrafish^28,29^ (Fig. 2a) are equally affected.

### Hematopoietic abnormalities in *dnmt1* mutant offspring

**Fig. 3:**
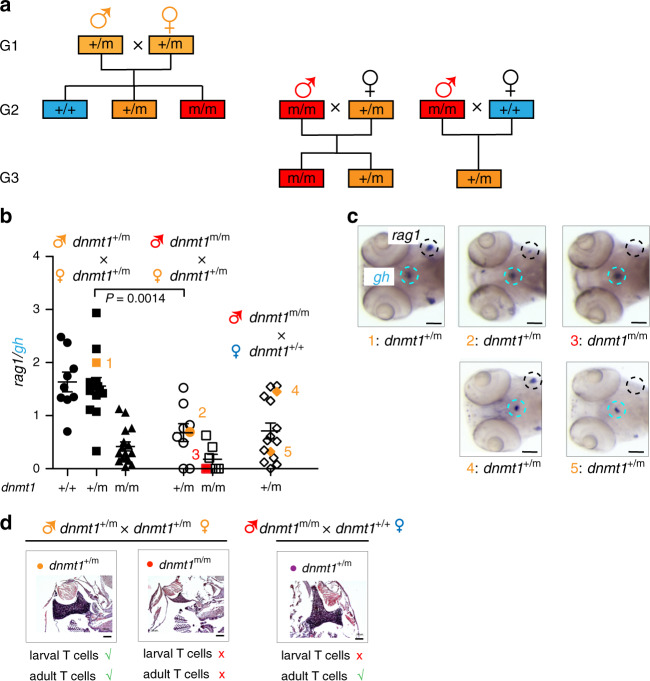
Impaired T cell development in 5 dpf larvae. **a** Structure of pedigrees. A cross of male and female carriers gives rise to the three genotypes of the G2 generation that are used to establish the G3 generation. **b** The *rag1/gh* ratio as determined by RNA in situ hybridization is shown for 5 dpf fish of the indicated genotypes (bottom) arising from the indicated parental genotypes (top); male genotypes are shown in upper row. Each data point represents one animal; *t* test, two-tailed; mean ± s.e.m. **c** Representative RNA in situ patterns on which the calculation of *rag1/gh* ratios was done are shown on the right for the animals identified by numbers in panel (**b**). The thymus region (identified by *rag1* signal) and the hypohysis (identified by the *gh* signal) are encircled; because of the cell-type specificity of the mutant phenotype, the *rag1/gh* ratio serves as a convenient normalization measure for the RNA in situ hybridization protocol. **d** Thymopoiesis in adult animals of the indicated generations and genotypes. Histological sections of thymi were stained with hematoxylin/eosin; note the alymphoid thymus in *dnmt1*^m/m^ fish. In (**c**, **d**), scale bars: 0.1 mm. Source data are provided as Source Data file.

We found that the offspring (designated as generation 3 [G3] in Fig. 3a) of male mutant fish crossed with wild-type females are viable, as are those of male mutant fish crossed with heterozygous females. Therefore, it was possible to examine the hematopoietic compartments of larval and adult stages of G3 fish. In contrast to heterozygous G2 animals arising from a cross of heterozygous males and females, we observed that many of the heterozygous G3 animals resulting from a cross of homozygous mutant males with wild-type females (Fig. 3a) exhibited impaired larval T cell development (Fig. 3b, c). By contrast, the adult T cell compartment of these heterozygous G2 and G3 fish develops normally (Fig. 3d). The striking discrepancy between the hematopoietic phenotypes of G2 and G3 fish suggests that the functionally distinct phases of T cell development in zebrafish are not only genetically^28,29^, but also epigenetically separable. Moreover, the present results suggest a particular sensitivity of larval T cell development to aberrations of DNA methylation.

**Fig. 4:**
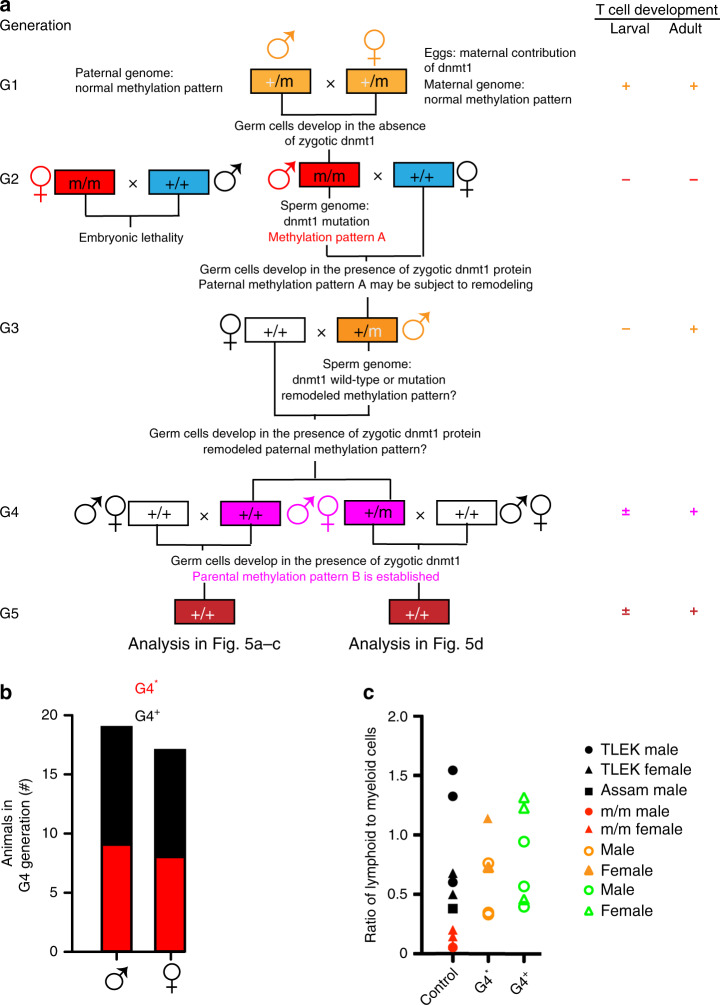
Transgenerational inheritance of failing larval T cell development. **a** Structure of pedigree; the genotypes and sex of animals is indicated. White boxes, wild-type animals unrelated to the *dnmt1*-mutant lineage; blue boxes, genotypically wild-type animals arising from various crosses in the *dnmt1*-mutant lineage. The presence (+) or absence (−) of larval and adult phases of T cell development in different generations of the pedigree are indicated on the right, with colors matching the relevant genotypes. The ± sign indicates that larval T cell development is impaired in some but not all G4 and G5 animals. **b** Genotypically wild-type (*dnmt1*^+/+^) males and females of the G4 generation give rise to off-spring with (G4*) or without (G4^+^) impaired larval T cell development when crossed to wild-type animals. **c** Normal adult hematopoiesis in fish of different genetic backgrounds. Whole kidney marrow cells were analyzed by flow cytometry (see Fig. 2c, left panel) and cell numbers in the lymphoid and myeloid gates were counted^47^. The genotype of animals is indicated to the right; TLEK and Assam represent wild-type strains. Note the normal lymphoid/myeloid ratios in adult G4* animals. Each data point represents one animal. Source data are provided as Source Data file.

The unexpected finding of impaired larval T cell development in heterozygous fish arising from crosses of mutant males and wild-type females prompted us to extend the analysis to include further generations (Fig. 4a). To this end, the G1 mutant carrier was out-crossed to wild-type fish twice, to establish two separate pedigrees. The extent of larval and adult T cell development was examined at each generation, but no differences were observed between these two separate populations. We found that in contrast to the situation of G2 mutant males, the progeny of a cross of G2 mutant females with wild-type males succumbed to an early developmental arrest before body axis formation. During early development, the maternal methylome is gradually reprogrammed to the paternal methylation pattern^13–15,30^, indicating that the enzymatic activity of the mutant Dnmt1 protein deposited in the eggs is not sufficient to support this remodeling. Because full Dnmt1 activity appears to be required before the onset of zygotic gene activation, only mutant males could be used to establish heterozygous G3 progeny; these fish were crossed to wild-type fish to generate *dnmt1*^+/+^ and *dnmt1*^+/m^ animals of the G4 generation (Fig. 4a). When we crossed male or female fish of the G4 generation that are genotypically wildtype for *dnmt1* with wild-type females or males (Fig. 4a), G5 fish with strikingly reduced larval T cell development were observed in ~50% of the crosses (Fig. 4b); this phenotype was observed in both pedigrees, arguing against the fortuitous co-segregation of an unknown mutation. Henceforth, G4 fish that yield normal offspring are designated as G4^+^, whereas those that sire offspring with impaired larval T cell development are designated as G4*. As expected, the cellular composition of the hematopoietic cell populations in the kidney marrow was normal for genotypically wild-type adult males and females that gave rise to offspring with impaired larval T cell development (Fig. 4c).

### Transgenerational inheritance of larval T cell impairment

**Fig. 5:**
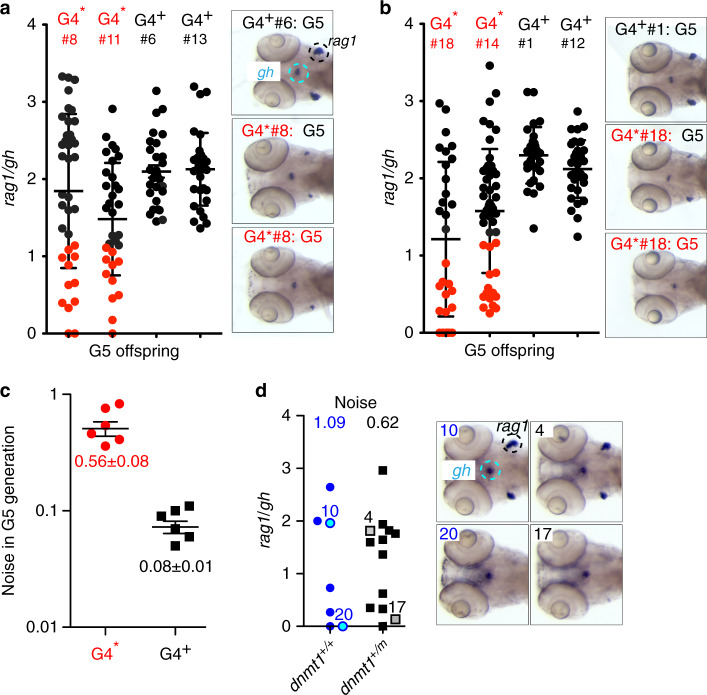
Variable levels of larval T cell development in the G5 generation. **a** Extent of T cell development (expressed as *rag1/gh* ratio) in the G5 generation arising from *dnmt1*^+/+^ male G4 fish (c.f., **a**). Red dots represent values of thymopoietic index of animals in G5 clutches arising from a G4* parent whose *rag/gh* ratios lie outside 2 standard deviations of values observed in G5 clutches arising from G4^+^ parents. Representative RNA in situ results are shown for G5 fish. **b** Extent of T cell development in the G5 generation arising from *dnmt1*^+/+^ female G4 fish. **c** Noise (calculated as variance/mean) values of *rag1/gh* data in G5 clutches arising from G4* and G4^+^ parents (mean ± s.e.m.).**d** Extent of T cell development (expressed as *rag1/gh* ratio) in the G5 generation arising from *dnmt1*^+/m^ male G4* fish; the mean noise values are indicated at the top (left panel). Representative RNA in situ patterns on which the calculation of *rag1/gh* ratios was performed are shown on the right for the animals identified by numbers in the left panel. In (**a**–**d**), each data point represents one animal. Source data are provided as Source Data file.

In G4* x wild-type crosses, ~1/3 of fish of the clutch presented with T cell deficiency; this phenomenon was observed in both male (Fig. 5a) and female (Fig. 5b) G4 animals. Indeed, when the range of *rag1*/*gh* values observed in the resulting G5 off-spring is determined and expressed as “noise” (variance normalized to the mean), G4^+^ and G4* animals can be clearly distinguished; G4* animals exhibit a 7-fold higher noise in *rag1/gh* values (Fig. 5c). The phenomenon of reduced larval T cell development in genotypically wild-type G5 fish was also seen in crosses of *dnmt1*^+/m^ G4 males with wild-type females, again accompanied by increased noise in the *rag1*/*gh* ratios in the two resulting genotypes (Fig. 5d). Collectively, these data suggest that the transgenerational inheritance of an aberrant DNA methylation mark(s) may underlie the aberrant larval T cell development.

In order to exclude the possibility that this phenomenon is the result of non-specific demethylation of the parental genomes, we repeated the crosses using wild-type parents treated with 5-aza-2′-deoxcytidine (aza-dC), an irreversible inhibitor of DNA methylases^31^. However, while aza-dC treatment reduced the extent of larval T cell development, no abnormalities were observed at later stages (Supplementary Fig. 1); most importantly, the adults exposed to the demethylating agent during the embryonic period did not produce offspring with aberrant T cell development, suggesting that the particular nature of changes in methylation patterns associated with the N1391K mutation underlies the transgenerational phenotype.

### Characterization of DNA methylation patterns in G4 sperm

Next, we analyzed the methylation patterns of G2 wild-type and mutant sperm. In the DNA of G2 *dnmt1*^m/m^ sperm, we detected 223,956 hypomethylated and 175 hypermethylated differentially methylated regions (DMRs); for simplicity, we refer to the methylation pattern in G2 mutant sperm as pattern A. Interestingly, G2 fish homozygous for the *dnmt1* mutation exhibit increased expression levels of the de novo DNA methyltransferase *dnmt3bb.2* at 5 dpf, suggesting the activation of compensatory mechanism(s) partially counteracting impaired maintenance activity of dnmt1; only minor differences were noted for other genes encoding modulators of DNA methylation (Supplementary Fig. 2a).

**Fig. 6:**
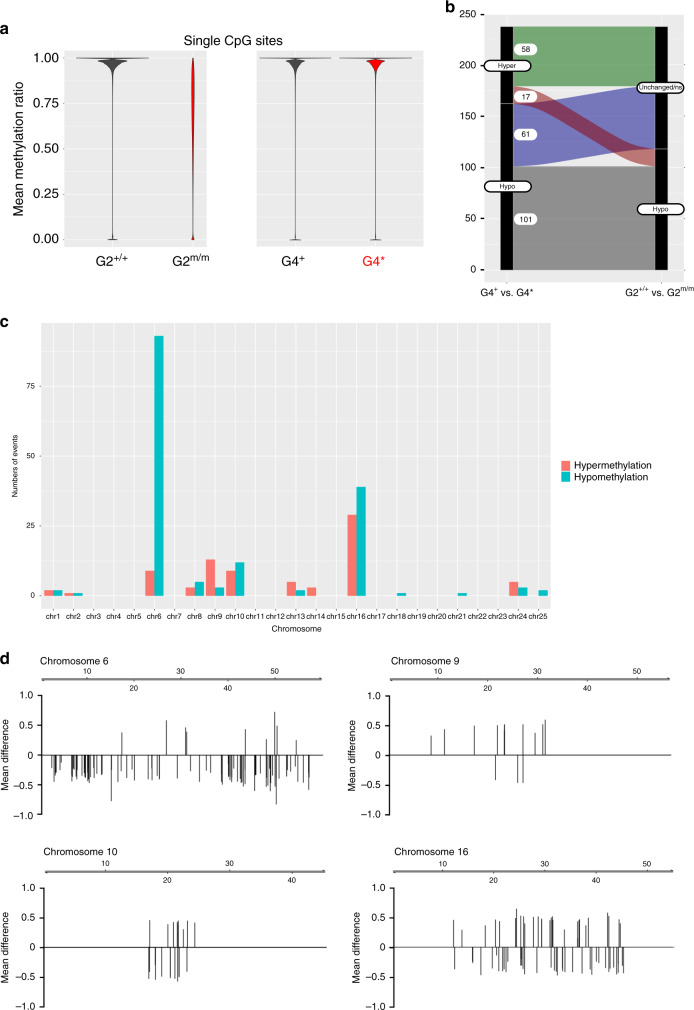
Dynamic changes of DNA methylation patterns in the G4 generation. **a** Mean methylation ratios of CpG dinucleotides in sperm DNAs of G2 *dnmt1*^+/+^ and *dnmt1*^m/m^ animals (left panel), and genotypically wild-type (*dnmt1*^+/+^) males of the G4 generation giving rise to off-spring with (G4*) or without (G4^+^) impaired larval T cell development when crossed to wild-type females (right panel). **b** History of methylation of DMRs distinguishing G4^+^ and G4* animals. The y-axis gives the number of DMRs that are either hypermethylated or hypomethylated in sperm DNA of in G4* versus G4^+^ animals. These DMRs were either indistinguishable in sperm of the G2 generation or were hypomethylated in G2^m/m^ versus G2^+/+^ animals. **c**, **d** Non-random distribution of DMRs in the genome of sperm of G4* relative to G4^+^ animals. Most DMRs represent hypomethylated sites **c**. Distribution of DMRs across the 4 most affected chromosomes (scale in Mb); the mean methylation differences are indicated; positive values indicate hypermethylation of DMRs in G4* sperm, negative values indicate hypomethylation of DMRs in G4^*^ sperm (**d**). Source data are provided as Source Data file.

We then analyzed the DNA methylation patterns of sperm of the two groups of genotypically wild-type G4 males, G4^+^ and G4*, respectively, and found that they differed by a mere 243 DMRs; 164 DMRs are hypomethylated in G4* (Supplementary Data 1), and 79 DMRs are hypermethylated in G4* sperm (Supplementary Data 2); we refer to the methylation patterns in G4 mutant sperm as patterns B^+^ and B*, respectively. For for G4^+^ sperm, the degree of methylation of CpG dinucleotides across the genomes is 97.57% (median; mad 2.99%); for G4*, this value is 97.53% (median; mad 3.02%) (Fig. 6a); these methylation levels are very close to those of wild-type sperm (Fig. 1f). The vast majority of DMRs identified in the comparison of G2^+/+^ and G2^m/m^ sperm could also be evaluated in G4 sperm. Of the 222,449 hypomethylated G2 DMRs, the methylation levels of 222,390 DMRs (99.3%) were identical between G4^+^ and G4* sperm DNAs; of the 171 hypermethylated G2 DMRs, 170 DMRs (99.4%) were no longer distinguishable in G4 sperm. Collectively, our observations suggest that during the transition from G2 to G4, the germ cells developing in the presence of zygotically provided dnmt1 protein undergo global restoration of methylation patterns.

Next, we examined the history of the DMRs distinguishing G4^+^ and G4* sperm DNAs (Fig. 6b). Of the 164 hypomethylated DMRs, 162 DMRs were also observed in the G2 comparison and could thus be included in our analysis; 101 DMRs were still hypomethylated in G4* sperm, whereas a further 61 DMRs that were originally indistinguishable in G2 exhibit lower methylation levels in G4* (Fig. 6b; Supplementary Data 3). Of the 79 hypermethylated DMRs distinguishing G4^+^ and G4* sperm DNAs, the status of 75 DMRs could be assessed in G2; 58 of these DMRs exhibited similar methylation levels in wild-type and mutant G2 sperm, whereas 17 DMRs were found to have lower methylation levels in G2^m/m^ sperm as compared to wildtype (Fig. 6b; Supplementary Data 3). The DMRs distinguishing G4^+^ and G4* sperm DNAs are non-randomly distributed across the genome (Fig. 6c), with chromosomes 6, 9, 10, and 16 being particularly affected (Fig. 6d).

### Gene regulatory context of DMRs

Next, we wished to gain sight into the distribution of DMRs in G2 and G4 with respect to known gene regulatory elements. We found that CpG islands, promoters, exons, introns and intergenic regions were similarly affected by the extensive changes in methylation of G2 mutant sperm DNA. Moreover, none of these features was associated with the methylation differences observed between G4^+^ and G4* sperm DNAs (Supplementary Fig. 2b). As expected, genomic regions marked by placeholder nucleosomes^15^ were found to be mostly unmethylated, and no changes in methylation patterns at these sites were observed in any of the experimental groups. Moreover, DMRs exhibiting dynamic changes during early zebrafish development, such as those changing between the epiboly stage and the 24 hpf time point, and those changing between the 24 hpf time point and the 48 hpf time point^32^ also could not distinguish G4^+^ and G4* sperm DNAs (Supplementary Fig. 2c). The analysis of repetitive sequences indicated that all classes of repeats were uniformly affected by hypomethylation in G2 mutant sperm DNA; in G4 sperm, all repeats participated in the restoration of methylation levels equally well (Supplementary Fig. 2d).

We then asked whether any particular chromatin signature would explain the differences between the methylation patterns of G4^+^ and G4* sperm DNAs and examined the enrichment of specific chromatin marks at the hypo- and hypermethylated G4 DMRs. None of the distinct DNA methylation patterns could be explained by histone modification combinations (Supplementary Fig. 3). Since we observed a disproportionately high number of G4 DMRs on chromosome 6 (Fig. 6c, d), we investigated DMR distribution in context of chromosome-scale features. To this end, we compared chromosome 6 (large number of DMRs discovered) with chromosome 7 (no DMRs discovered) (Supplementary Fig. 4). We found that neither the positions of topologically associated domains (TADs), nor the presence of specific chromatin marks (H3K4me3; H3K27me3; H3K9me3), CpG islands, or repetitive sequences were associated with clusters of hypo- and hypermethylated DMRs (Supplementary Fig. 4). Further studies are required to determine the mechanistic basis of the uneven distribution of DMRs distinguishing the methylation patterns of G4^+^ and G4* sperm DNAs.

### Characterization of hypermethylated DMRs in G4* sperm

**Fig. 7:**
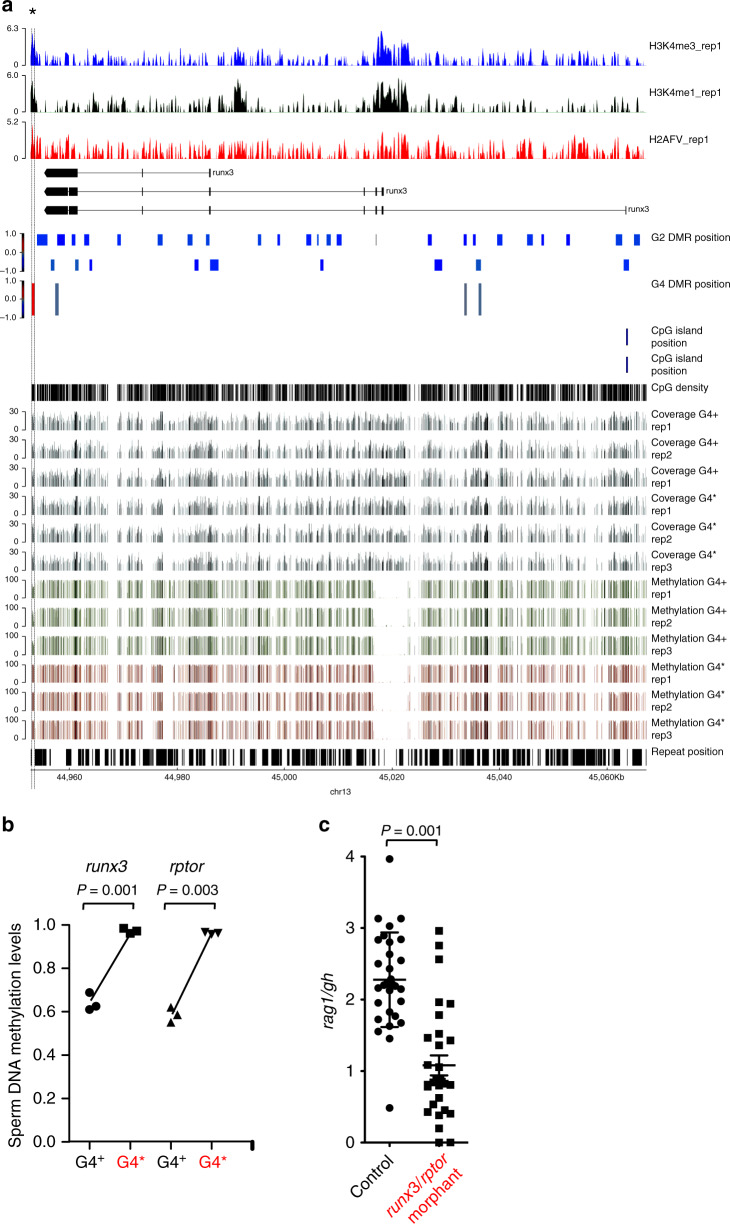
Molecular basis of transgenerational inheritance of impaired larval T cell development. **a** Characterization of the *runx3* locus. In the first three rows, various chromatin marks are indicated^15^. The structures of known transcripts across the *runx3* locus are shown below. The positions of candidate DMRs called in G2 sperm DNAs are shown underneath the transcript structures, as are the three candidate DMRs discovered in G4^+^ and G4* sperm DNA. The position of the only annotated *runx3* CpG island is indicated above the CpG density track. The filtered coverage across the locus in the three WGBS replicates is indicated as well as the extent of methylation in the CpGs that were evaluated in the comparison of G4^+^ and G4* sperm DNAs. The bottom row indicates the positions of repeat across the locus. Note that the hypermethylated DMR in G4* sperm DNA coincides with a peak in H3K4 methylation. **b** DNA methylation levels in DMRs associated with *runx3* and *rptor* genes in sperm of G4 males. For *runx3*, the data pertain to the three replicates of the DMR marked with an asterisk in a; for *rptor*, the relevant DMR is indicated by an asterisk in Supplementary Fig. 5b. **c** Combined knock-down of *runx3* and *rptor* by anti-sense oligonucleotides impairs larval T cell development. Each data point represents one animal analyzed at 5 dpf by RNA in situ hybridization. In (**b**, **c**), each data point represents one animal; t-test, two-tailed; mean ± s.e.m. Source data are provided as Source Data file.

Our results suggest that the hematopoietic abnormalities of G2 mutants have their origin in a progenitor common to both T and B lymphoid lineages in larvae and adult fish. By contrast, G4* animals give rise to a more circumscribed aberration in their offspring, originating in the incapacitation or lack of a larval T cell progenitor only. This in turn suggests that the failure of T cell development observed in G4* progeny may have a different or only partially overlapping epigenetic basis than that in earlier generations. Hence, we focused our attention on those DMRs in G4 sperm whose methylation levels are discordant to G2 mutant sperm (Fig. 6b; Supplementary Data 3). To this end, we searched for DMRs associated with genes known to be important for T cell development. Notably, in G4* sperm, we identified one hypermethylated DMR each that is associated with *runx3* and *rptor* genes, respectively (Fig. 7a, b; Supplementary Fig. 5a, b); previous studies in mice have shown that these genes are both required for intrathymic T cell development^33,34^. In both cases, the hypermethylated DMR overlaps with a region distinguished by chromatin marks, such as H3K4me1, H3K4me3, and H2AFV, indicative of regulatory function(s)^15^. Although we consider it likely that genes other than *runx3* and *rptor* and/or their concerted activities contribute to the observed T cell phenotype in the offspring of G4* animals, we found that simultaneous knock-down of these two genes led to impaired larval T cell development (Fig. 7c). Moreover, despite the fact that the expression of *runx3* and *rptor* are not exclusive to the T cell lineage, a trend towards lower expression levels of both genes was observed in 5 dpf mutant larvae (Supplementary Fig. 5c). Collectively, our findings suggest that *runx3* and *rptor* are functionally relevant for zebrafish T cell development and that their epigenetic regulation contributes to the transgenerational phenomenon observed here.

## Discussion

The present study documents a rare example of transgenerational inheritance of epimutations in vertebrates, and, to the best of our knowledge, the first case of such a phenomenon that is linked with a specific immunological phenotype. When mutant sperm fertilize an egg produced by wild-type or *dnmt1*^+/m^ heterozygous females, developmentally programmed changes of DNA methylation patterns during germ cell development occur in the presence of maternally provided dnmt1 protein. Paternal methylomes are known to be stably propagated throughout embryogenesis, and the heavily methylated sperm DNA determines the DNA methylome of the early embryo^13–15^. However, remodeling of an aberrant paternal methylation pattern can still occur, otherwise the low levels of methylation observed in mutant sperm would have been preserved over the course of several generations. As a result of the combined activities of dnmt1 and other dnmts, such as those with de novo activities, as well as other pathways modulating the DNA methylation pattern in direct or indirect fashion, nearly complete restoration of the original wild-type methylation pattern is achieved in G4 sperm. Hence, it appears that the developmentally programmed changes in DNA methylation patterns are controlled by a rheostat that is blind to the origin of the parental genomes, supporting the notion that the paternal methylation pattern does not serve as a template for the remodeling of the maternal methylome^13^. Our results also indicate that the partially restored methylation pattern appears to exist in two subtly different forms; one of these segregating epi-alleles fails to support larval T cell development, the other is seemingly compatible with normal lymphopoiesis. Overall, the differences in DNA methylation between G4^+^ and G4* sperm appear limited; in analogy to the situation in plants^35,36^, it may be possible to use epigenetic inbred lines to precisely identify the crucial epigenetic signature that supports larval T cell development. Unfortunately, at present, methods for the purification of zebrafish lymphoid precursors are not available. Once this becomes possible, it will be interesting to compare the methylation patterns of gametes with those of somatic cells giving rise to the specific phenotype observed here.

Our results support the notion that lymphoid development is particularly sensitive to perturbation of DNA methylation; this phenomenon is not restricted to fish, since the phenotype of impaired lymphopoiesis is reminiscent of that of mice with low expression levels of wild-type protein^37,38^. Collectively, these findings suggest that DNA methylation serves as a mechanism to protect the development of lymphoid cells^39^. Since lymphocytes represent an evolutionarily recent innovation in the haematopoietic system^40^, it attests to the flexibility of epigenetic regulation to integrate evolutionary novelties into pre-existing physiology. Certain immunodeficiency syndromes in animals and humans are caused by genetic alterations of DNMTs^41–43^ or are linked to sporadic epigenetic variations^44^. However, given the differences in the processes of parental reprogramming between fish and mammals, we consider it improbable that the mechanism underlying the particular immunodeficiency disorder described here also applies to so far unexplained immunodeficiency disorders in human patients.

## Methods

### Animals

The zebrafish (*D. rerio*) wild-type strain TLEK (Tüpfel long fin/Ekkwill) is maintained in the animal facility of the Max Planck Institute of Immunobiology and Epigenetics, Freiburg, Germany and was used for crosses with the *dnmt1* mutants. The IY071 mutant line has been described^23^, as has been the *ikaros:eGFP* transgenic reporter line^27,28^. All animal experiments were performed in accordance with relevant guidelines and regulations, approved by the review committee of the Max Planck Institute of Immunobiology and Epigenetics and the Regierungspräsidium Freiburg, Germany (license Az 35-9185.81/G-14/106).

### Morphants

Morphants were generated by injection of anti-sense morpholino oligonucleotides (Gene Tools, Philomath, OR) to block translation of both maternal and zygotic mRNAs (“ATG morpholinos”), or to block splicing of zygotic pre-mRNAs (“splice morpholinos”). To block zygotic *rptor* activity, a morpholino targeting the splice donor site of exon 3 of the *rptor* gene was used (5´- TGGATGGATGGATGCTCACCTATC; final concentration in injection buffer 0.067 mM); to inhibit translation of *runx3* mRNA, a morpholino overlapping the initiation site was used (5´- ACGGGAATATGCATCACAACAGATT; final concentration in injection buffer 0.133 mM). Stock solutions of morpholinos were diluted as required in injection buffer (0.05% (v/v) phenol red; 1x Danieau Buffer (http://cshprotocols.cshlp.org/content/2011/7/pdb.rec12467.full). Approximately 1–2 nL of solution were injected into fertilized eggs^23^. The morphants were analyzed at 5 dpf by RNA in situ hybridization using a combination of *rag1*- and *gh*-specific probes, and the results expressed as a thymopoietic index, a dimensionless number (see below).

### Thymopoietic index

Thymic *rag1* gene expression is a marker of ongoing assembly of T cell receptor genes. Hence, the intensity of the RNA in situ signal correlates with the number of differentiating T cells, which we consider to be a measure of T cell development. In order to provide an internal control (technical, with respect to the hybridization process as such; and, biological, with respect to the tissue specificity of the observed genetic effects), we employed a probe specific for the growth hormone (*gh*) gene, which marks a subset of cells in the hypophysis. Determination of *rag1/gh* ratios was carried out as follows: after RNA in situ hybridization with *rag1* and *gh* probes, ventral images of 4–5 dpf zebrafish larvae were taken on an MZFLIII (Leica) microscope using a digital camera DFC300FX (Leica), essentially generating a two-dimensional projection of the three-dimensional structure. The areas of *rag1* and *gh* signals were measured using ImageJ (ImageJ 1.52a; available at http://imagej.nih.gov/ij), and the ratio of average of the *rag1*-positive area vs. *gh*-positive area was calculated as a measure of thymopoietic activity. After photographic documentation of the RNA in situ hybridization signal, larvae were processed for genomic DNA extraction for subsequent genotyping.

### RNA extraction and cDNA synthesis

Total RNA was extracted using TRI Reagent (Sigma) following the manufacturer’s instructions. After treatment with DNaseI (Promega), RNA extraction using TRI Reagent was repeated. Superscript II Reverse Transcriptase (Invitrogen) and oligo(dT) were used for cDNA synthesis from total RNA.

### Quantitative PCR

qPCR was carried out using SYBR Premix Ex Taq (Takara) and 7500 fast real-time PCR system (Applied Biosystems)^23,45^. *actb1* was used as a reference gene. The primer sets for zebrafish genes were purchased from BioRad (https://www.bio-rad.com/de-de/product/primepcr-pcr-primers-assays-arrays?ID=M0HROA15). *gata2b*, qDreCID0018645; *cmyb*, qDreCID0021456; *mpx*, qDreCID0017849; *gata1a*, qDreCID0013676; *cebpa*, qDreCED0006492; *ikzf1*, qDreCID0018943; *gata3*, qDreCED0006945; *rag1*, qDreCED0021315; *lck*, qDreCID0022016; *zap70*, qDreCID0014002; *dnmt1*, qDreCED0019976; *dnmt3aa*, qDreCID0018392; *dnmt3ab*, qDreCID0019082; *dnmt3ba* (aka *dnmt3b*), qDreCED0021338; *dnmt3bb.1* (aka *dnmt4*), qDreCID0005035; *dnmt3bb.2* (aka *dnmt3*), qDreCID0016654; *dnmt3bb.3* (aka *dnmt5*), qDreCED0021863; *gadd45aa*, qDreCED0006281; *gadd45ab*, qDreCED0015505; *tet1*, qDreCED0015074; *tet2*, qDreCED0010969; *tet3*, qDreCID0016164; *actb1*, qDreCED0020462.

### Histological analysis

Histological analysis was carried out after formalin fixation, paraffin embedding and hematoxylin/eosin staining of sections^46^. For image analysis of histological sections, JmageJ software was used (ImageJ 1.52a; available at http://imagej.nih.gov/ij).

#### Flow cytometry

Flow cytometric analysis of light-scatter characteristics of WKM cells was carried out as described by Traver et al.^47^; dead cells were excluded by staining with FluoroGold (Santa Cruz).

### Treatment of embryos with Dnmt inhibitors

A stock solution of 5-Aza-2′-deoxycytidine (Sigma) was prepared in E3 medium, and diluted to the desired final concentration. Wild-type embryos were exposed to the inhibitor^48^ beginning at 24 hpf for a total of 48 h; at 72 hpf, embryos were washed and continuously cultured in E3 medium.

### Whole genome bisulfite sequencing

Genomic DNA was extracted from three *dnmt1*^+/+^ and three *dnmt1*^−/−^ zebrafish of G2 generation at 18 dpf, sperm of three *dnmt1*^+/+^ and three *dnmt1*^−/−^ zebrafish of G2 generation, and sperm of three G4* and three G4+ fish of G4 generation using the DNeasy blood and tissue kit (Qiagen). 1 μg and 0.5 μg of DNA was used for bisulfite reactions and library construction using the TruSeq DNA PCR-free library preparation kit (Illumina) and the EpiGnome Methyl-Seq kit (Epicentre), respectively. The fragments were sequenced in paired-end 100 bp mode on 1 lane of Illumina HiSeq 2500 instrument.

### Whole genome bisulfite read alignment

Raw sequencing reads were trimmed with cutadapt version 1.9.1 ref. ^49^ as follows: the first two (TruSeq) or six (Epignome) 5′-most nucleotides were hard-trimmed and Illumina adapter sequences removed. Bisulfite-specific operations on reads and reference genome were performed with methylCtools version 0.9.4 ref. ^50^. Bisulfite-converted reads were mapped to bisulfite-converted danRer10 zebrafish genome with bwa-mem version 0.7.12 separately for the two library types. Back-converted bam files were sorted with samtools version 1.3.1, PCR duplicates removed and read group information added with Picard tools v1.136. The two resulting bam files per sample were merged with samtools and methylation bias profiled with MethylDackel v0.1.7 [https://github.com/dpryan79/MethylDackel]. Bam files from this step were further used as inputs for de novo DMR discovery in each dataset, as well as for evaluation of methylation values of target genomic regions provided as bed files (for details, see below).

### Extraction of methylation values per CpG

Methylated and unmethylated read counts per CpG position were extracted with methylCtools v0.9.4 with mapping quality threshold of 10, SNP detection, counting only one of two overlapping paired end reads, skipping 5 nucleotides from each read length and zero-padding of uncovered positions.

### Quality filtering of CpGs and data plotting

Data postprocessing was performed in R version 3.2.3. Raw methylation values were set to NA for CpG positions with at least 0.25 SNP allelic frequency as well as for positions with aggregate coverage of less than 10 reads. Mean methylation ratios per CpG position were calculated as mean over all the replicates per group. Only complete observations were used (positions with any NA values were removed). These group-mean CpG methylation ratios were used to produce density plots in Fig. 1.

### Detection of de novo methylated regions (DMRs)

Methylation values for single CpG positions (complete cases) were used as input to metilene v 0.2-6 ref. ^51^. Wild-type samples were passed in as group A, and mutant as group B. Candidate DMRs detected by metilene were re-evaluated for differential methylation in R. At least 20% of detected CpGs per DMR were required and at most 1 sample with an NA value was allowed. Methylation values of detected CpGs were aggregated as mean per interval per replicate sample. Differential methylation was re-evaluated using limma on logit-transformed interval means. DMRs were filtered to retain those with FDR <5%. Filtered DMRs were further annotated with the distance to the nearest gene using bedtools version 2-2.19.0 and Ensembl release 83 gene models for GRCz10.

### Generation of coverage and methylation bigwig files

Bigwig files with coverage and methylation for G2 and G4 datasets were generated by running snakePipes^52^ v1.2.1 with G2 and G4 bam files as input.

### Evaluation of G4 sperm DMRs in G2 sperm WGBS data

Bam files obtained through read alignment were re-analyzed with the WGBS workflow in the snakePipes^52^ v1.2.1 modified for the purpose of this manuscript (see, https://github.com/katsikora/snakepipes_fork). Methylation values were extracted from G2 bam files for genomic intervals identifed as hypo- and hypermethylated DMRs in the comparison of the G4* vs G4 + fish sperm, separately. Aggregate methylation values per interval were obtained as above for the DMRs. The matrix of logit-transformed methylation values per interval was input to differential analysis with limma version 3.26.9 ref. ^53^. Intervals were filtered for absolute difference of at least 20% between MT and WT fish and FDR <2%.

### Reanalysis of publically available zebrafish ChIP-seq data

Input and ChIP reads for zebrafish sperm H3K4me3, H3K4me1, H2AFV, H3K27ac, H3K27me3, H3K14ac^15^ were downloaded from GEO (GSE95033), as were input and ChIP reads for 6.0hpf zebrafish embryo H3K9me3^54^, GSE113086). This embryo stage was chosen as there was no sufficient H3K9me3 signal in the earlier embryo stages^54^. Reads were mapped to zebrafish genome danRer10 and processed with snakePipes^52^ version 1.3.0 DNA-mapping and ChIP-seq workflows to produce bigwig files with log2 ratio ChIP over input.

### Reanalysis of publically available zebrafish embryo HiC data

HiC reads for 24hpf embryos^55^ were downloaded from GEO (GSE105013) and processed with snakePipes 1.3.0 HiC workflow to produce HiC matrices at 20 kb resolution and to call TAD positions.

### *runx3* and *raptor* genomic tracks plots

Genomic tracks plot was obtained with pyGenomeTracks^56^ version 3.2. Used were log2 ratio ChiP over input bigwig files obtained for H3K4me3, H3K4me1 and H2AFV marks as described above, Ensembl version 88 gene model gtf for GRCz10, bed files with G2 and G4 DMR positions, bed file with CpG island position for danRer10 (UCSC), bed file with positions of all CpGs in the reference genome (“CpG density”), bigwig files with filtered CpG coverage for G4+ and G4* replicates, bigwig files with methylation value (0–100%) for G4+ and G4* replicates, and a repeat masker bed file for danRer10 (UCSC). Dashed vertical lines highlight the position of the single DMR differentially methylated (hypermethylated) in G4* vs G4+ fish. The *runx3* plot was generated for genomic interval chr13:44952862–45067319 and the *rptor* plot for the genomic interval chr6:17422590–17702769, covering the gene locus and adding 3 kb flanking regions on each side of it.

### Methylation values for gene regulatory elements

Genomic intervals for gene regulatory features were obtained with the genFeatures function of the BioConductor R package systemPipeR^57^ v1.6.2. Input was Ensembl release 83 gtf file and GRCz10 chromosome sizes. The genome was segmented into promoters, introns, exons, and intergenic regions. Promoters were defined as 500nt upstream to transcriptional start site, irrespectively of any positionwise overlap with another feature. The other three feature types were further reduced to disjoint ranges i.e. nonoverlapping any other feature, and of length at least 5nt. CpG island positions for danRer10 from UCSC were used as is. To obtain genomic intervals for placeholder nucleosomal regions, H2AZ and H3K4me1 ChIP-seq peaks^15^ downloaded as bed files from GEO (GSE95030) were intersected. Intersecting regions of at least 100nt length were used in further analysis. Bed files with genomic intervals specified above were input to modified snakePipes WGBS v.1.2.1 workflow, and evaluated in both G2 and G4 datasets to obtain mean methylation ratios per interval and violin plots.

### Methylation values for developmental DMRs

Genomic positions for zebrafish developmental DMRs (24hpf vs epiboly, 48hpf vs 24hpf, hypo- and hypermethylated) were downloaded from Supplementary materials of the original publication^32^ and lifted-over to danRer10 genome using the UCSC lift-over web tool. DMR lists were input to snakePipes v.1.2.1 and evaluated in both G2 and G4 datasets to obtain mean methylation ratios per interval and violin plots.

### Methylation values for zebrafish genomic repeats

Repeat masker file for danRer10 from UCSC was converted to bed file, and used as input to snakePipes v.1.2.1 to evaluate in both G2 and G4 datasets and obtain mean methylation ratios per interval and density plots. Methylation extraction for genomic repeats was done with the same threshold on mapping quality as for the other target intervals (at least 10).

### Coincidence of DMRs with chromosome-level genomic features

Genomic tracks for chromosomes 6 and 7 were produced with pyGenomeTracks^56^ version 3.2. Used were HiC matrices for 24hpf embryo and bed file with TAD positions for 24hpf embryo obtained as described in sections above, log2 ratio ChiP over input bigwig files obtained for H3K4me3, H3K27me3 and H3K9me3 marks as described in sections above, bigwig file with filtered CpG coverage for one G4 replicate, bed files with hypo- and hypermethylated G4 DMR positions, bed file with CpG positions (UCSC) and bed file with repeat positions (UCSC). Dashed vertical lines show TAD boundaries obtained as described in sections above.

### Coincidence of histone modification with G4 DMRs

Bigwig files of log2 ratio ChiP over input obtained for H3K3me1, H3K4me3, H3K27me3, H2AFV, and H3k14ac for merged replicates were analyzed with deepTools v3.3.1. ChIP signal aggregated over 10 nt bins was extracted for unchanged, hypo- and hypermethylated G4 DMRs, rescaled to 500 nt with 100 nt added on each flank, to produce a matrix with DMRs in rows and bins in columns, grouped by chromatin mark and DMR source. Profile plots of log2 ratio ChIP signal over input with 0.95 confidence intervals were obtained for the DMR groups using deepStats v0.4 ref. ^58^ with 1000 bootstraps, as were binwise Wilcoxon-test P value plots. A cutoff adjusted *P* value of 0.05 was used.

### Statistical methods

No randomization of animals was done in the present studies; phenotypes were recorded by a blinded observer before genotyping. No animals were excluded from analyses. Samples size was estimated from the degrees of variability in previous analyses^23,46^ in order to be able to detect biologically meaningful differences in examined parameters, usually 20% difference from control values. t-tests were performed for samples with equal variance; otherwise, F-tests were used. Other statistical procedures are detailed in the respective sections above.

### Reporting summary

Further information on research design is available in the Nature Research Reporting Summary linked to this article.

## Supplementary information

Supplementary Information

Descriptions of Additional Supplementary Files

Supplementary Data 1

Supplementary Data 2

Supplementary Data 3

Supplementary Code

Reporting Summary

## Data Availability

The original sequencing data have been deposited in the GEO database and are available under accession number “GSE98647”. All other relevant data supporting the key findings of this study are available within the article and its Supplementary Information files or from the corresponding author upon reasonable request. Source data are provided with this paper. A reporting summary for this Article is available as a Supplementary Information file. Source data are provided with this paper.

## Source data

Source Data

## Code availability

The R code necessary to reproduce the statistical analyses and results is reported in Supplemental Code and is available at https://github.com/katsikora/Iwanami2019_SupplementaryCodeAndData_A.
