# Supplementary material for: Transgenerational inheritance of impaired larval T cell development in zebrafish
Source: Nat Commun. 2020 Sep 9;11:4505. doi: 10.1038/s41467-020-18289-9 (PMC7481223; doi:10.1038/s41467-020-18289-9)
Supplement: Supplementary file 1 — Supplementary Information [file 41467_2020_18289_MOESM1_ESM.pdf]

Supplementary Information

Transgenerational inheritance of impaired larval T cell development in zebrafish

Norimasa Iwanami, Divine-Fondzenyuy Lawir, Katarzyna Sikora, Connor O´Meara, Kohei Takeshita, Michael Schorpp, & Thomas Boehm

This file includes:

Supplementary Figures 1-5
Legends for Supplementary Data 1-3
Supplementary references

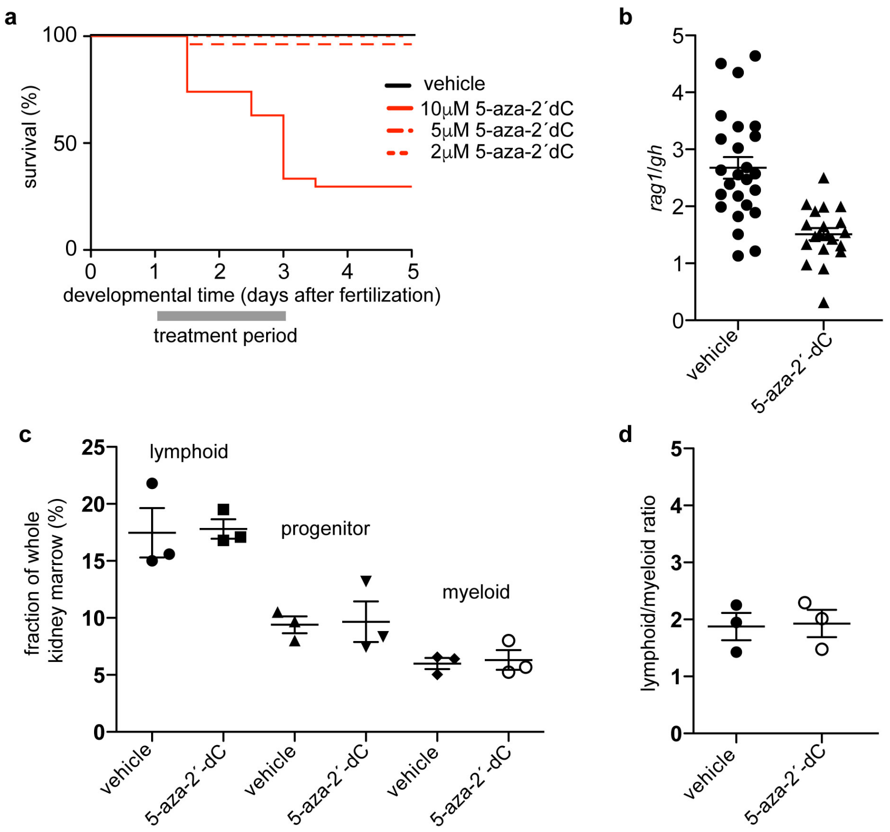

Supplementary Fig. 1 Phenotype of zebrafish treated with a methyltransferase inhibitor. **a** Survival curve of fish (n=27) treated with 5-aza-2´-deoxycytdine (5-aza-2´-dC) at various concentrations; note that >95% of embryos treated with 5-aza-2´-deoxycytidine at a final concentration of 5 mM survived. **b** Impaired T cell development in 5 dpf larvae treated with 5-aza-2´-deoxycytidine at 5 mM, as in **a**. **c, d** Normal adult haematopoiesis in fish treated with 5-aza-2´-deoxycytidine at 5 mM during the larval period, as in **a**, **b**. Whole kidney marrow cells of age-matched animals were analysed by flow cytometry, and the fractions of total cells in the respective gates determined (**c**), and the ratio of lymphoid and myeloid cells calculated (**d**). Offspring of crosses of treated adult fish with wildtype fish do not exhibit impaired larval T cell development; note that values of the lymphoid/myeloid ratio are age-dependent. In c-d, mean±s.e.m. values are indicated. Source data are provided as Source Data file.

a

Gene	*dnmt1*$^{+/+}$	*dnmt1*$^{-/-}$	P	n	Gene	*dnmt1*$^{+/+}$	*dnmt1*$^{-/-}$	P	n
dnmt1	1.00±0.09	0.90±0.11	0.5038	4	*gadd45aa*	1.00±0.12	0.90±0.12	0.5779	4
dnmt3aa	1.00±0.17	0.41±0.01	0.0133	4	*gadd45ab*	1.00±0.10	0.91±0.14	0.6057	4
dnmt3ab	1.00±0.08	0.56±0.05	0.0041	4	*tet1*	1.00±0.04	0.78±0.02	0.0085	3
dnmt3ba	1.00±0.04	1.11±0.15	0.5051	4	*tet2*	1.00±0.03	0.69±0.02	0.0006	3
dnmt3bb.1	1.00±0.04	0.94±0.07	0.4749	4	*tet3*	1.00±0.05	0.80±0.03	0.0270	3
dnmt3bb.2	1.00±0.07	2.36±0.11	<0.0001	4					
dnmt3bb.3	1.00±0.08	1.00±0.11	0.9859	4					

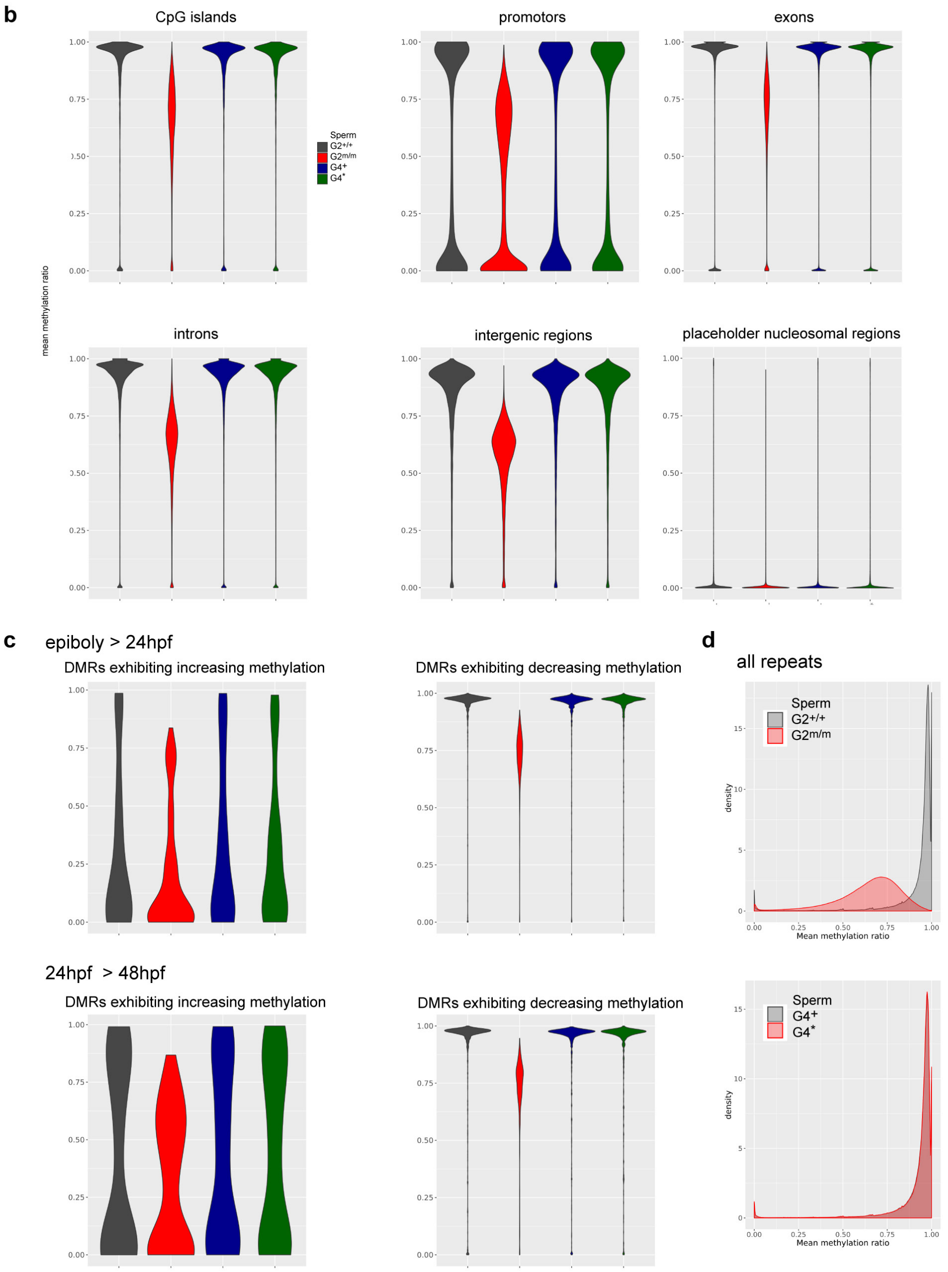

Supplementary Fig. 2 Characteristics of G2 and G4 generations. **a** Gene expression patterns in G2 larvae of the indicated genotypes normalized to wild-types (t-test; two-tailed). **b-d** Extent of DNA methylation in sperm DNA of G2 wild-type and homozygous mutants, and G4+ and G4* animals with respect to genomic features. The DMRs exhibiting dynamic changes during development in **c** were taken from Ref. 1. Source data are provided as Source Data file.

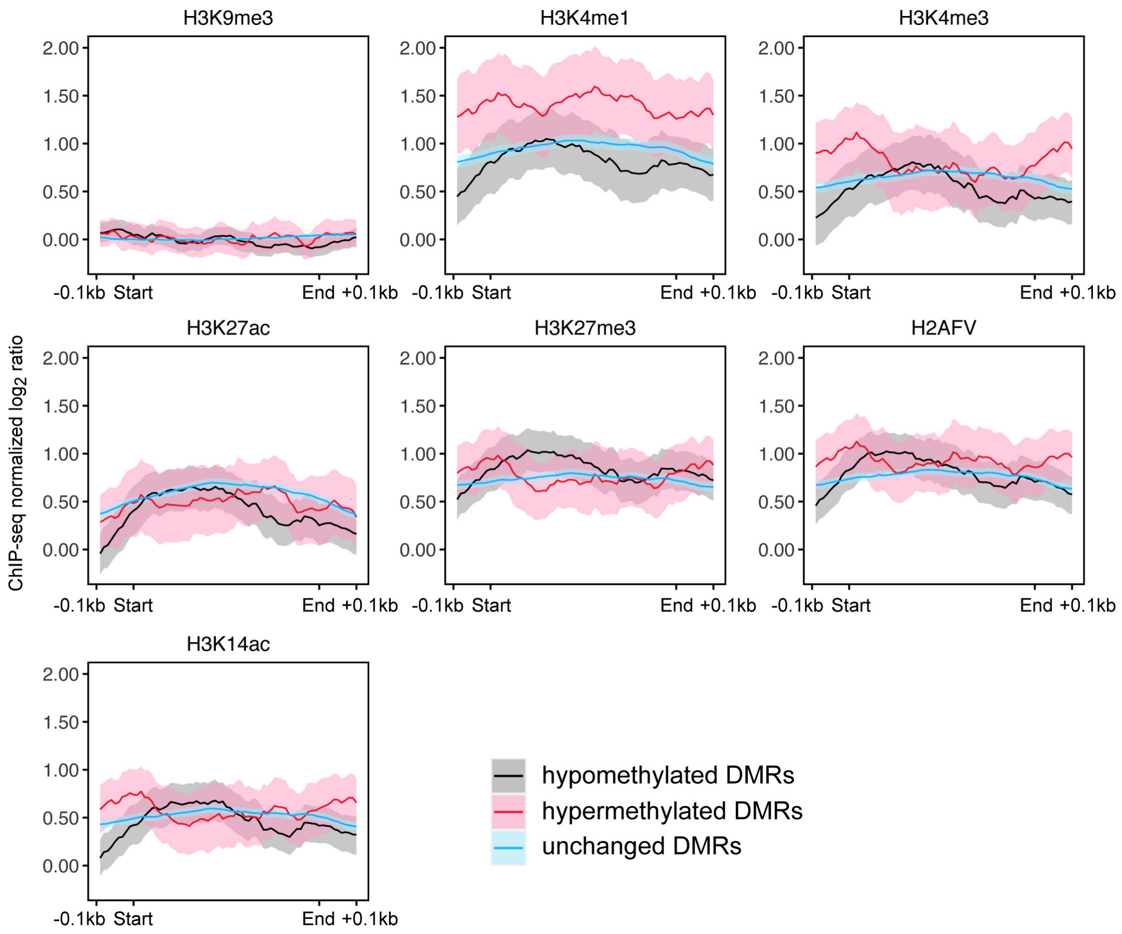

Supplementary Fig. 3 Profile plots of ChIP-seq signal over input with mean (solid line) and 95% confidence intervals for G4 DMR groups (G4* versus $G4^+$ sperm DNA). The profiles for unchanged, hypomethylated, and hypermethylated. DMRs are indicated by different colours. Data for chromatin marks taken from Ref. 2 (H3K9me3) and from Ref. 3 (remaining marks). Statistical analysis (see Methods) revealed that for all DMR intervals, the differences between the three categories of DMRs did not reach statistical significance at $P<0.05$. Source data are provided as Source Data file.

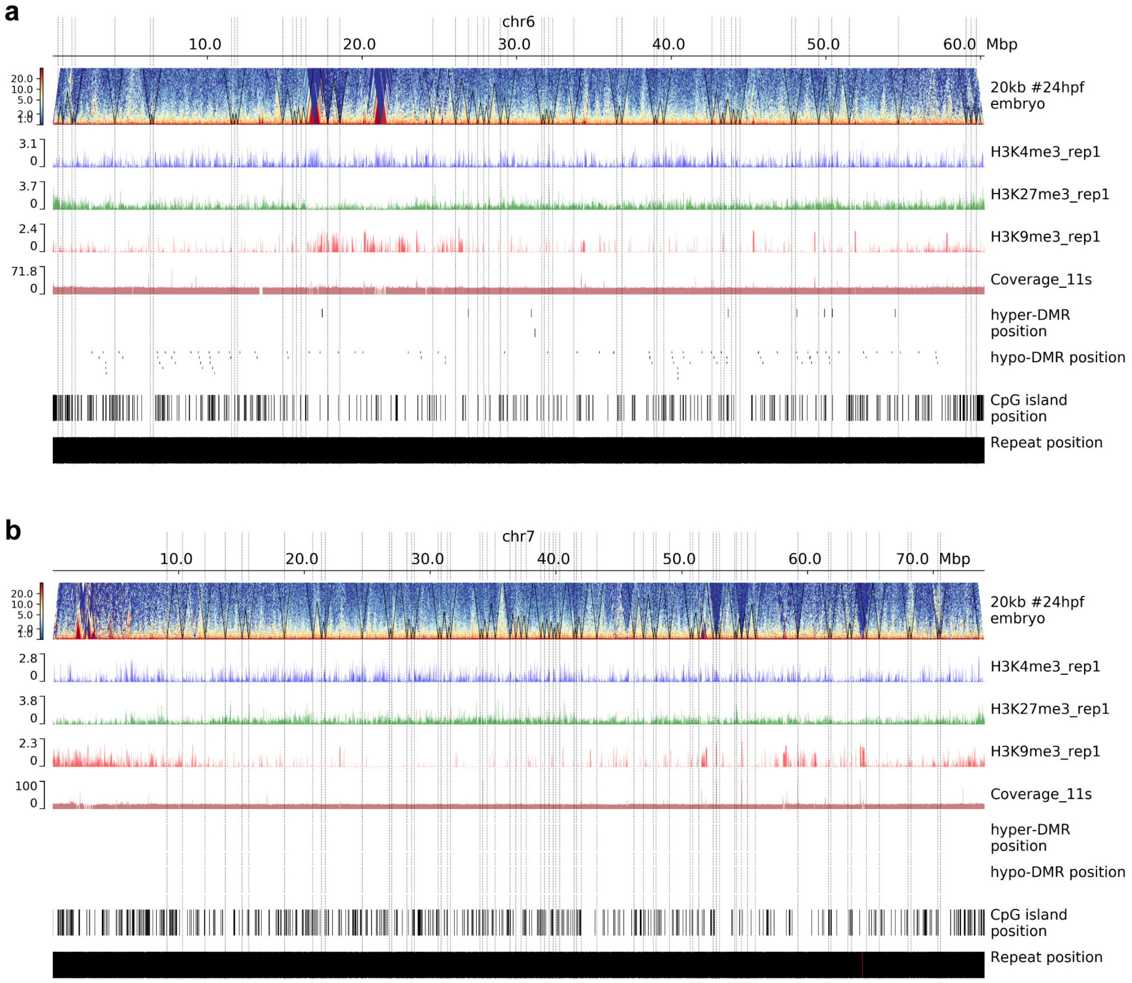

Supplementary Fig. 4 Lack of correlation between chromosome-level chromatin features and positions of DMRs in G4 sperm. **a** TAD boundaries for chromosome 6, aligned with the indicated chromatin marks, coverage of WGBS experiments and positions of DMRs (cf., Fig. 6d) presented here, together with positions of CpG islands and repeats. **b** Features as in a for chromosome 7. Data for TADs were taken from ref. 4, chromatin marks taken from ref. 3. Source data are provided as Source Data file.

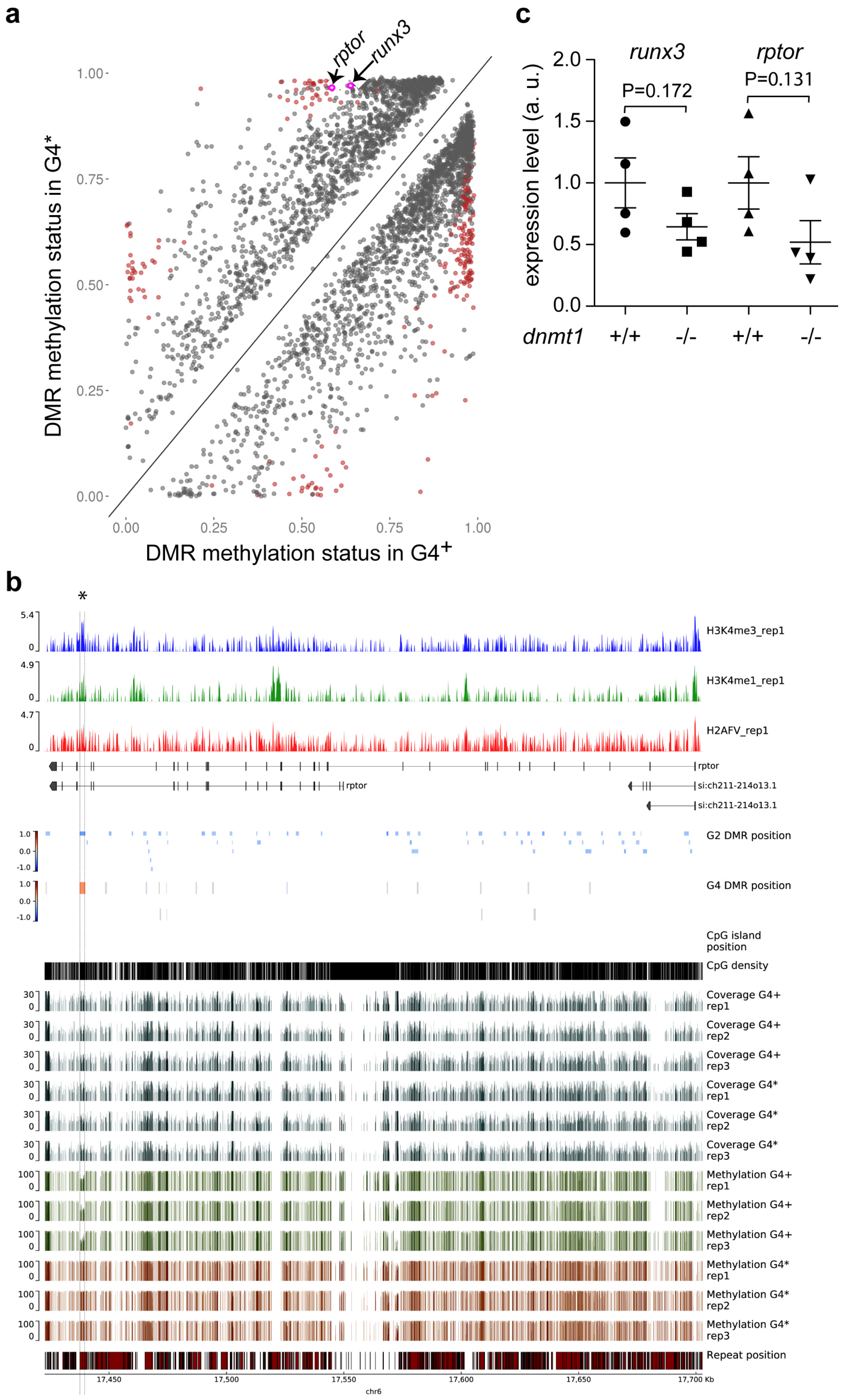
a
rptor
runx3
DMR methylation status in G4*
DMR methylation status in G4+
1.00
0.75
0.50
0.25
0.00
c
expression level (a. u.)
runx3
rptor
P=0.172
P=0.131
2.0
1.5
1.0
0.5
0.0
dnmt1
+/+
-/-
+/+
-/-
b
*
H3K4me3_rep1
H3K4me1_rep1
H2AFV_rep1
rptor
si:ch211-214o13.1
G2 DMR position
G4 DMR position
CpG island position
CpG density
Coverage G4+ rep1
Coverage G4+ rep2
Coverage G4+ rep3
Coverage G4* rep1
Coverage G4* rep2
Coverage G4* rep3
Methylation G4+ rep1
Methylation G4+ rep2
Methylation G4+ rep3
Methylation G4* rep1
Methylation G4* rep2
Methylation G4* rep3
Repeat position
17,450
17,500
17,550
17,600
17,650
17,700 Kb
chr6

Supplementary Fig. 5 Molecular basis of transgenerational inheritance of impaired larval T cell development. **a** G4 DMR pairwise DNA methylation in G4+ and G4* groups. DMRs passing the thresholds on statistical parameters are indicated in red. **b** Characterization of the *rptor* locus. In the first three rows, various chromatin marks are indicated[3]. The structures of known transcripts across the *rptor* locus are shown below. The positions of DMRs distinguishing G2 sperm DNAs are shown underneath the transcript structures, as are the three DMRs distinguishing $G4^{+}$ and G4* sperm DNAs. The empty CpG island track indicates no annotated CpG islands for this locus. CpG density track is shown. The coverage across the locus in the three WGBS replicates is indicated as well as the extent of methylation in the CpGs that were evaluated in the comparison of $G4^{+}$ and G4* sperm DNAs. The bottom row indicates the positions of repeat across the locus. Note that the hypermethylated DMR in G4* sperm DNA coincides with a peak in H3K4 methylation. **c** Reduced expression levels of *runx3* and *rptor* genes in 5 dpf larvae (n=4, t-test, two-tailed; mean±s.e.m.). Source data are provided as Source Data file.

Legends to Supplementary Data files

Supplementary Data 1. Hypomethylated differentially methylated regions distinguishing sperm DNA of G4* and $G4^+$ males. Chr, chromosome; Start/Stop, coordinates of differentially methylated region (DMR); N.CG., number of CpG dinucleotides in DMR; G4+, mean methylation level in DMR in non-transmitting fish; G4*, mean methylation level in DMR in transmitting fish; adj.P.Val, adjusted P value from limma analysis; qvalue, significance score from metilene analysis; ENSDARG, Ensembl gene identifier; Distance, distance in nucleotides from transcription start site (0, DMR in the body of the gene; -, DMR upstream of gene; +, DMR downstream of gene). The calculation of extents of methylation was carried out as described in Methods, Detection of *de novo* methylated regions (DMRs). The original sequencing data have been deposited in the GEO database and are available under accession number GSE98647.

Supplementary Data 2. Hypermethylated differentially methylated regions distinguishing sperm DNA of G4* and $G4^+$ males. For column designation, see Supplementary Table 1. The calculation of extents of methylation was carried out as described in Methods, Detection of *de novo* methylated regions (DMRs). The original sequencing data have been deposited in the GEO database and are available under accession number GSE98647.

Supplementary Data 3. Differentially methylated regions in G4 sperm grouped according to methylation status in G2 mutant sperm. For column designation, see Supplementary Table 1. The calculation of extents of methylation was carried out as described in Methods, Detection of *de novo* methylated regions (DMRs). The original sequencing data have been deposited in the GEO database and are available under accession number GSE98647.

Supplementary references

1. Bogdanovic, O. et al. Active DNA demethylation at enhancers during the vertebrate phylotypic period. *Nat. Genet.* **48**, 417-426 (2016).
2. Laue, K., Rajshekar, S., Courtney, A. J., Lewis, Z. A. & Goll, M. G. The maternal to zygotic transition regulates genome-wide heterochromatin establishment in the zebrafish embryo. *Nat. Commun.* **10**, 1551 (2019).
3. Murphy, P. J., Wu, S. F., James, C. R., Wike, C. L. & Cairns, B. R. Placeholder Nucleosomes Underlie Germline-to-Embryo DNA Methylation Reprogramming. *Cell* **172**, 993-1006 e1013 (2018).
4. Kaaij, L. J. T., van der Weide, R. H., Ketting, R. F. & de Wit, E. Systemic Loss and Gain of Chromatin Architecture throughout Zebrafish Development. *Cell Rep.* **24**, 1-10 e14 (2018).
