# Supplementary material for: Transgenerational inheritance of impaired larval T cell development in zebrafish
Source: Nat Commun. 2020 Sep 9;11:4505. doi: 10.1038/s41467-020-18289-9 (PMC7481223; doi:10.1038/s41467-020-18289-9)
Supplement: Supplementary file 2 — Descriptions of Additional Supplementary Files [file 41467_2020_18289_MOESM2_ESM.pdf]

Descriptions of Additional Supplementary Files

Supplementary Data 1.

Description: Hypomethylated differentially methylated regions distinguishing sperm DNA of G4* and G4+ males.

Supplementary Data 2.

Description: Hypermethylated differentially methylated regions distinguishing sperm DNA of G4* and G4+ males.

Supplementary Data 3.

Description: Differentially methylated regions in G4 sperm grouped according to methylation status in G2 mutant sperm
