# Supplementary material for: Transgenerational inheritance of impaired larval T cell development in zebrafish
Source: Nat Commun. 2020 Sep 9;11:4505. doi: 10.1038/s41467-020-18289-9 (PMC7481223; doi:10.1038/s41467-020-18289-9)
Supplement: Supplementary file 7 — Reporting Summary [file 41467_2020_18289_MOESM7_ESM.pdf]

nature research

Corresponding author(s): Thomas Boehm

Last updated by author(s): July 13, 2020

Reporting Summary

Nature Research wishes to improve the reproducibility of the work that we publish. This form provides structure for consistency and transparency in reporting. For further information on Nature Research policies, see Authors & Referees and the Editorial Policy Checklist .

Statistics

For all statistical analyses, confirm that the following items are present in the figure legend, table legend, main text, or Methods section.

n/a	Confirmed	
☐	☒	The exact sample size (*n*) for each experimental group/condition, given as a discrete number and unit of measurement
☐	☒	A statement on whether measurements were taken from distinct samples or whether the same sample was measured repeatedly
☐	☒	The statistical test(s) used AND whether they are one- or two-sided<br>*Only common tests should be described solely by name; describe more complex techniques in the Methods section.*
☒	☐	A description of all covariates tested
☐	☒	A description of any assumptions or corrections, such as tests of normality and adjustment for multiple comparisons
☐	☒	A full description of the statistical parameters including central tendency (e.g. means) or other basic estimates (e.g. regression coefficient) AND variation (e.g. standard deviation) or associated estimates of uncertainty (e.g. confidence intervals)
☐	☒	For null hypothesis testing, the test statistic (e.g. *F*, *t*, *r*) with confidence intervals, effect sizes, degrees of freedom and *P* value noted<br>*Give P values as exact values whenever suitable.*
☒	☐	For Bayesian analysis, information on the choice of priors and Markov chain Monte Carlo settings
☐	☒	For hierarchical and complex designs, identification of the appropriate level for tests and full reporting of outcomes
☒	☐	Estimates of effect sizes (e.g. Cohen's *d*, Pearson's *r*), indicating how they were calculated

Our web collection on statistics for biologists contains articles on many of the points above.

Software and code

Policy information about availability of computer code

Data collection	The code used to analyze the data is summarized at https://github.com/katsikora/Iwanami2019_SupplementaryCodeAndData_A
Data analysis	Whole genome bisulfite read alignment. Raw sequencing reads were trimmed with cutadapt version 1.9.1 (Ref. 39) as follows: the first two (TruSeq) or six (Epignome) 5'-most nucleotides were hard-trimmed and Illumina adapter sequences removed. Bisulfite-specific operations on reads and reference genome were performed with methylCtools version 0.9.4 (Ref. 40). Bisulfite-converted reads were mapped to bisulfite-converted GRCz10 zebrafish genome with bwa-mem version 0.7.12 separately for the two library types. Back-converted bam files were sorted with samtools version 1.3.1, PCR duplicates removed and read group information added with Picard tools v1.136. The two resulting bam files per sample were merged with samtools and methylation bias profiled with MethylDackel v0.1.7 [https://github.com/dpryan79/MethylDackel].<br>Extraction of methylation values per CpG. Methylated and unmethylated read counts per CpG position were extracted with methylCtools v0.9.4 with mapping quality threshold of 10, SNP detection, counting only one of two overlapping paired end reads, skipping 5 nucleotides from each read length and zero-padding of uncovered positions.<br>Postprocessing and statistical analyses. Data postprocessing was performed in R version 3.2.3. Raw methylation values were set to NA for CpG positions with at least 0.25 SNP allelic frequency as well as for positions with aggregate coverage of less than 10 reads. Mean methylation ratios per CpG position were calculated as mean over all the replicates per group. Only complete observations were used (positions with any NA values were removed).<br>Detection of de novo methylated regions (DMRs). Methylation values for single CpG positions (complete cases) were used as input to metilene v 0.2-6 (Ref. 41). Wildtype samples were passed in as group A, and mutant as group B. DMRs detected by metilene were re-evaluated for differential methylation in R. At least 20% of detected CpGs per DMR were required and at most 1 sample with an NA value was allowed. Methylation values of detected CpGs were aggregated as mean per interval per replicate sample. Differential methylation was re-evaluated using limma on logit-transformed interval means. DMRs were filtered to retain those with FDR<5%. Filtered DMRs were further annotated with the distance to the nearest gene using bedtools version 2-2.19.0 and ensembl release 83 gene models for GRCz10.<br>Evaluation of G1 sperm DMRs in G4 sperm WGBS data. Bam files obtained through read alignment were re-analyzed with the WGBS

workflow in the snakePipes42 1.2.1 version modified to accommodate user-defined formula and contrast [https://github.com/katsikora/snakepipes_fork]. Methylation values were separately extracted for genomic intervals identified as hypo- and hyper-methylated DMRs in the comparison of the G1 mutant vs wild-type fish sperm. Aggregate methylation values per interval were obtained as above for the DMRs. The matrix of logit-transformed methylation values per interval was input to differential analysis with limma version 3.26.9 (Ref. 43). The following linear model was fit '~1+batch+condition:batch', where batch differentiated G1 and G4 samples, while condition differentiated treatment (G1 mutant and G4*) from control (G1 wildtype and G4+) samples. Finally, a topTable of differentially methylated intervals was extracted for the contrast 'batchG4_conditionTreatment-batchG1_conditionTreatment'. The numbers of intervals filtered with an absolute difference of at least 20% and FDR<2% were counted.

For image analysis, JmageJ software was used (ImageJ 1.52a; available at http://imagej.nih.gov/ij)

For manuscripts utilizing custom algorithms or software that are central to the research but not yet described in published literature, software must be made available to editors/reviewers. We strongly encourage code deposition in a community repository (e.g. GitHub). See the Nature Research guidelines for submitting code & software for further information.

Data

Policy information about availability of data

All manuscripts must include a data availability statement. This statement should provide the following information, where applicable:

- Accession codes, unique identifiers, or web links for publicly available datasets
- A list of figures that have associated raw data
- A description of any restrictions on data availability

The R code necessary to reproduce the statistical analyses and results presented in Fig. 1e,f; Fig. 4,a-c; Extended Data Figure 3 a,b,c; Extended Data Table 2; Extended Data Table 3 is reported in Supplemental Code available at The R code necessary to reproduce the statistical analyses and results is reported in Supplemental Code and available at https://github.com/katsikora/Iwanami2019_SupplementaryCodeAndData_A. The original sequencing data have been deposited in the GEO database and are available under accession number GSE98647; this includes GSM2607706, GSM2607707, GSM2607708, GSM2607709, GSM2607710, GSM2607711 ,GSM2607712,
GSM2607713, GSM2607714, GSM2607715, GSM2607716, GSM2607717, GSM4125287, GSM4125288, GSM4125289, GSM4125290, GSM4125291, GSM4125292, GSM4125293, GSM4125294, GSM4125295, GSM4125296, GSM4125297, GSM4125298, GSM4125299, GSM4125300, GSM4125301, GSM4125302, GSM4125303, GSM4125304, GSM4125305, GSM4125306, GSM4125307, GSM4125308, GSM4125309, GSM4125310.Source data are provided with this paper.

Field-specific reporting

Please select the one below that is the best fit for your research. If you are not sure, read the appropriate sections before making your selection.

☒ Life sciences ☐ Behavioural & social sciences ☐ Ecological, evolutionary & environmental sciences

For a reference copy of the document with all sections, see nature.com/documents/nr-reporting-summary-flat.pdf

Life sciences study design

All studies must disclose on these points even when the disclosure is negative.

Sample size	Samples size was estimated from the degrees of variability in previous analyses (Iwanami, N. et al. Genetic evidence for an evolutionarily conserved role of IL-7 signaling in T cell development of zebrafish. J. Immunol. 186, 7060-7066 (2011); Iwanami, N. et al. Forward genetic screens in zebrafish identify pre-mRNA-processing pathways regulating early T cell development. Cell Rep. 17, 2259-2270 (2016); Iwanami et al., Epigenetic Protection of Vertebrate Lymphoid Progenitor Cells by Dnmt1. iScience. 2020 Jun 10;23(7):101260. doi: 10.1016/j.isci.2020.101260), in order to be able to detect biologically meaningful differences in examined parameters, usually 20% difference from control values. This criterion required to include at least 3 animals for DNA methylome and gene expression analyses, and at least 6 animals for rag1/gh ratio determination.<br>t-tests were performed for samples with equal variance; otherwise, F-tests were used.
Data exclusions	No data were excluded
Replication	Biological replicates were conducted and the results are detailed in the manuscript; the results of replicate experiments were in agreement.
Randomization	When animals of the same genotype were used, they were randomly assigned to control and experimental groups
Blinding	Experimenters were blinded with respect to genotypes when analyzing phenotypes, since genotypes were determined only later.

Reporting for specific materials, systems and methods

We require information from authors about some types of materials, experimental systems and methods used in many studies. Here, indicate whether each material, system or method listed is relevant to your study. If you are not sure if a list item applies to your research, read the appropriate section before selecting a response.

Materials & experimental systems

n/a	Involved in the study	
☒	☐	Antibodies
☒	☐	Eukaryotic cell lines
☒	☐	Palaeontology
☐	☒	Animals and other organisms
☒	☐	Human research participants
☒	☐	Clinical data

Methods

n/a	Involved in the study	
☒	☐	ChIP-seq
☐	☒	Flow cytometry
☒	☐	MRI-based neuroimaging

Animals and other organisms

Policy information about studies involving animals; ARRIVE guidelines recommended for reporting animal research

Laboratory animals	zebrafish; for analyses at 5 dpf, sex cannot be determined
Wild animals	laboratory strains of zebrafish (Danio rerio) were used (TLEK [Tüpfel long fin/Ekkwill])
Field-collected samples	No field-collected animals were used in this study.
Ethics oversight	All animal experiments were performed in accordance with relevant guidelines and regulations, approved by the review committee of the Max Planck Institute of Immunobiology and Epigenetics and the Regierungspräsidium Freiburg, Germany (license Az 35-9185.81/G-14/106).

Note that full information on the approval of the study protocol must also be provided in the manuscript.

Flow Cytometry

Plots

Confirm that:

☒ The axis labels state the marker and fluorochrome used (e.g. CD4-FITC).

☒ The axis scales are clearly visible. Include numbers along axes only for bottom left plot of group (a 'group' is an analysis of identical markers).

☒ All plots are contour plots with outliers or pseudocolor plots.

☒ A numerical value for number of cells or percentage (with statistics) is provided.

Methodology

Sample preparation	Flow cytometric analysis of light-scatter characteristics of whole kidney marrow cells was carried out as described36; dead cells were excluded by staining with FluoroGold (Santa Cruz).
Instrument	FACS Aria
Software	For image analysis, JmageJ software was used (ImageJ 1.52a; available at http://imagej.nih.gov/ij)
Cell population abundance	as detailed in Fig. 1g
Gating strategy	FSC/SSC was used; lymphoid and myeloid gates are indicated in Fig. 2c; GFP fluorescence for lymphoid gates is also shown. The relevant gates are indicated in Fig. 2c; the lymphoid gate was analysed for GFP fluorescence and is indicated by the arrow in Fig. 2c.

☐ Tick this box to confirm that a figure exemplifying the gating strategy is provided in the Supplementary Information.
