# Supplementary material for: Transgenerational inheritance of impaired larval T cell development in zebrafish
Source: Nat Commun. 2020 Sep 9;11:4505. doi: 10.1038/s41467-020-18289-9 (PMC7481223; doi:10.1038/s41467-020-18289-9)
Supplement: Supplementary file 8 — Source Data [file 41467_2020_18289_MOESM8_ESM.zip › 19-35959B_source data file/source data list.docx]

Iwanami et al., Transgenerational inheritance of impaired larval T cell development in zebrafish

Source Data file (Part 1)

This sheet lists the primary data files used to generate the indicated display items. See also the **Supplementary Code** associated with this paper.

- Figure 1e: “<https://owncloud.gwdg.de/index.php/s/yukRgs4sCCaIJBh/download>” (zebrafish larvae single CpG methylation values),
- Figure 1f and Figure 6a left panel: “<https://owncloud.gwdg.de/index.php/s/rnhnQNXbsT6MyuK/download>” (zebrafish sperm single CpG methylation values), data for Fig. 1e and Fig. 1f are summarized in Fig. 1g.
- Figure 6a right panel: “<https://owncloud.gwdg.de/index.php/s/yBqvW1OFSA57lC9/download>” (zebrafish G4 sperm single CpG methylation values),
- Figure 6b: “<https://owncloud.gwdg.de/index.php/s/sHTiST0yjvMEXv6/download>” (zebrafish G4 sperm unchanged candidate DMRs re-evaluation in G2 sperm, RData format); “<https://owncloud.gwdg.de/index.php/s/IC1SivxF5Kh3xmH/download>” (zebrafish G4 sperm hypomethylated candidate DMRs re-evaluation in G2 sperm, RData format); “<https://owncloud.gwdg.de/index.php/s/gKeTusEW2RphUII/download>” (zebrafish G4 sperm hypermethylated candidate DMRs re-evaluation in G2 sperm, RData format),
- Figure 6c-d: “<https://owncloud.gwdg.de/index.php/s/IllgF5WJh6nQbWj/download>” (hypomethylated DMRs in zebrafish G4 sperm); “<https://owncloud.gwdg.de/index.php/s/sycC2qgeLFWsGsL/download>” (hypermethylated DMRs in zebrafish G4 sperm),
- Figure 7b: “<https://owncloud.gwdg.de/index.php/s/SwxeMrLXVXz43Jq/download>” (zebrafish G4 sperm DMR methylation values, RData format); “<https://owncloud.gwdg.de/index.php/s/aPaBBsfTQ9Mh72U/download>” (zebrafish G4 sperm DMR topTable, text format); this file is also related to Fig. 7a and Supplementary Fig. 5b.
- Supplementary Figure 2b: “<https://owncloud.gwdg.de/index.php/s/xM4iV7c0uBdbYtq/download>” (zebrafish CpG island methylation values in G2 and G4 zebrafish sperm, RData format); “<https://owncloud.gwdg.de/index.php/s/mdoY3EjAOJoM32X/download>” (zebrafish promoter methylation values in G2 and G4 zebrafish sperm, RData format); “<https://owncloud.gwdg.de/index.php/s/AReo7EfJbRhKIbC/download>” (zebrafish exon methylation values in G2 and G4 zebrafish sperm, RData format); “<https://owncloud.gwdg.de/index.php/s/uYQ5Yl8587aI2Ng/download>” (zebrafish intron methylation values in G2 and G4 zebrafish sperm, RData format); “<https://owncloud.gwdg.de/index.php/s/ap1hCKnBcZc0CkP/download>” (zebrafish intergenic region methylation values in G2 and G4 zebrafish sperm, RData format); “<https://owncloud.gwdg.de/index.php/s/tHtigelOj443mnT/download>” (zebrafish placeholder nucleosomal region methylation values in G2 and G4 zebrafish sperm, RData format),
- Supplementary Figure 2c: “<https://owncloud.gwdg.de/index.php/s/SFlvlC2XlBs8ioO/download>” (Bogdanovic 2016 24hpf vs epiboly hypermethylated DMR methylation values in G2 and G4 zebrafish sperm, RData format); “<https://owncloud.gwdg.de/index.php/s/5RJ5NKdeGd6zCzT/download>” (Bogdanovic 2016 24hpf vs epiboly hypomethylated DMR methylation values in G2 and G4 zebrafish sperm, RData format); “<https://owncloud.gwdg.de/index.php/s/o9WDVlqu977sTjF/download>” (Bogdanovic 2016 48hpf vs 24hpf hypermethylated DMR methylation values in G2 and G4 zebrafish sperm, RData format); “<https://owncloud.gwdg.de/index.php/s/0IbEcVV3SD6K2qo/download>” (Bogdanovic 2016 48hpf vs 24hpf hypomethylated DMR methylation values in G2 and G4 zebrafish sperm, RData format),
- Supplementary Figure 2d: “<https://owncloud.gwdg.de/index.php/s/Z1Rab7KdJWdGJEK/download>” (zebrafish genomic repeats methylation values in G2 zebrafish sperm, RData format); “<https://owncloud.gwdg.de/index.php/s/mYs5jyKUg5b7Kf4/download>” (zebrafish genomic repeats methylation values in G4 zebrafish sperm, RData format)
- Supplementary Figure 5a: “<https://owncloud.gwdg.de/index.php/s/m4f74VNi2hhK5LO/download>” (zebrafish G4 sperm group-mean DMR methylation values, RData format); “<https://owncloud.gwdg.de/index.php/s/KxVNGRREIavcA8O/download>” (zebrafish G4 sperm DMR topTable, RData format)

Data Source file (part 2)

The accompanying Excel document provides the raw data for the following display items

| Figure 2b, c | Sheet 1 |
| --- | --- |
| Figure 3b | Sheet 2 |
| Figure 4b, c | Sheet 3 |
| Figure 5a-d | Sheet 4 |
| Figure 7c | Sheet 5 |
| Supplementary Fig. 1 a-d | Sheet 6 |
| Supplementary Fig. 2a | Sheet 7 |
| Supplementary Fig. 5c | Sheet 8 |
